# *Flavobacterium petrolei* sp. nov., a novel psychrophilic, diesel-degrading bacterium isolated from oil-contaminated Arctic soil

**DOI:** 10.1038/s41598-019-40667-7

**Published:** 2019-03-11

**Authors:** Dhiraj Kumar Chaudhary, Dong-Uk Kim, Dockyu Kim, Jaisoo Kim

**Affiliations:** 1 Department of Life Science, College of Natural Sciences, Kyonggi University, Suwon, Gyeonggi-Do 16227 South Korea; 2 Division of Bio-convergence, College of Convergence and Integrated Science, Kyonggi University, Suwon, Gyeonggi-Do 16227 South Korea; 3 Division of Polar Life Sciences, Polar Research Institute, Incheon, 21990 South Korea

## Abstract

This study presents taxonomic description of two novel diesel-degrading, psychrophilic strains: Kopri-42^T^ and Kopri-43, isolated during screening of oil-degrading psychrotrophs from oil-contaminated Arctic soil. A preliminary 16S rRNA gene sequence and phylogenetic tree analysis indicated that these Arctic strains belonged to the genus *Flavobacterium*, with the nearest relative being *Flavobacterium psychrolimnae* LMG 22018^T^ (98.9% sequence similarity). The pairwise 16S rRNA gene sequence identity between strains Kopri-42^T^ and Kopri-43 was 99.7%. The DNA-DNA hybridization value between strain Kopri-42^T^ and Kopri-43 was 88.6 ± 2.1% indicating that Kopri-42^T^ and Kopri-43 represents two strains of the same genomospecies. The average nucleotide identity and *in silico* DNA-DNA hybridization values between strain Kopri-42^T^ and nearest relative *F*. *psychrolimnae* LMG 22018^T^ were 92.4% and 47.9%, respectively. These values support the authenticity of the novel species and confirmed the strain Kopri-42^T^ belonged to the genus *Flavobacterium* as a new member. The morphological, physiological, biochemical and chemotaxonomic data also distinguished strain Kopri-42^T^ from its closest phylogenetic neighbors. Based on the polyphasic data, strains Kopri-42^T^ and Kopri-43 represents a single novel species of the genus *Flavobacterium*, for which the name *Flavobacterium petrolei* sp. nov. is proposed. The type strain is Kopri-42^T^ (=KEMB 9005-710^T^ = KACC 19625^T^ = NBRC 113374^T^).

## Introduction

Anthropological activities have increased the level of oil-based contaminants in Arctic and Antarctica regions^1^. Psychrophilic bacteria have been implemented to remediate these hazardous oil-based pollutants from coldest regions of the Earth. Native psychrotrophs with degradation capabilities can be employed for xenobiotic transformation and bioremediation of petroleum hydrocarbons from Polar regions^2^. As a remediating agent, several strains of the genus *Flavobacterium* have been also used in the field of environmental bioremediation^3–5^.

The genus *Flavobacterium* belonging to the family *Flavobacteriaceae* of the phylum *Bacteroidetes* was first established by Bergey *et al*.^6^ and later its emended description was provided by Bernardet *et al*.^7^. During manuscript preparation, this genus accommodates 208 species with valid names (http://www.bacterio.net/flavobacterium.html). The members of the genus *Flavobacterium* comprises diverse habitat and have been isolated from compost materials, diseased fish, algal mat, marine environments, rhizospheric niches, toxic circumstances (including petroleum products-contaminated soil), and psychrophilic regions (glacier, Antarctic lake, Tibetan Plateau, and Arctic soil)^5–15^. During the search of psychrophilic degraders of petroleum products, strains Kopri-42^T^ and Kopri-43 were isolated from oil-contaminated Arctic soil. This study presents detail taxonomic investigation of strains Kopri-42^T^ and Kopri-43 and briefly illustrated their diesel-degrading capacity. On the basis of polyphasic taxonomic results, both the strains are proposed as novel members of the genus *Flavobacterium*, with Kopri-42^T^ as the type strain.

## Results and Discussion

**Figure 1:**
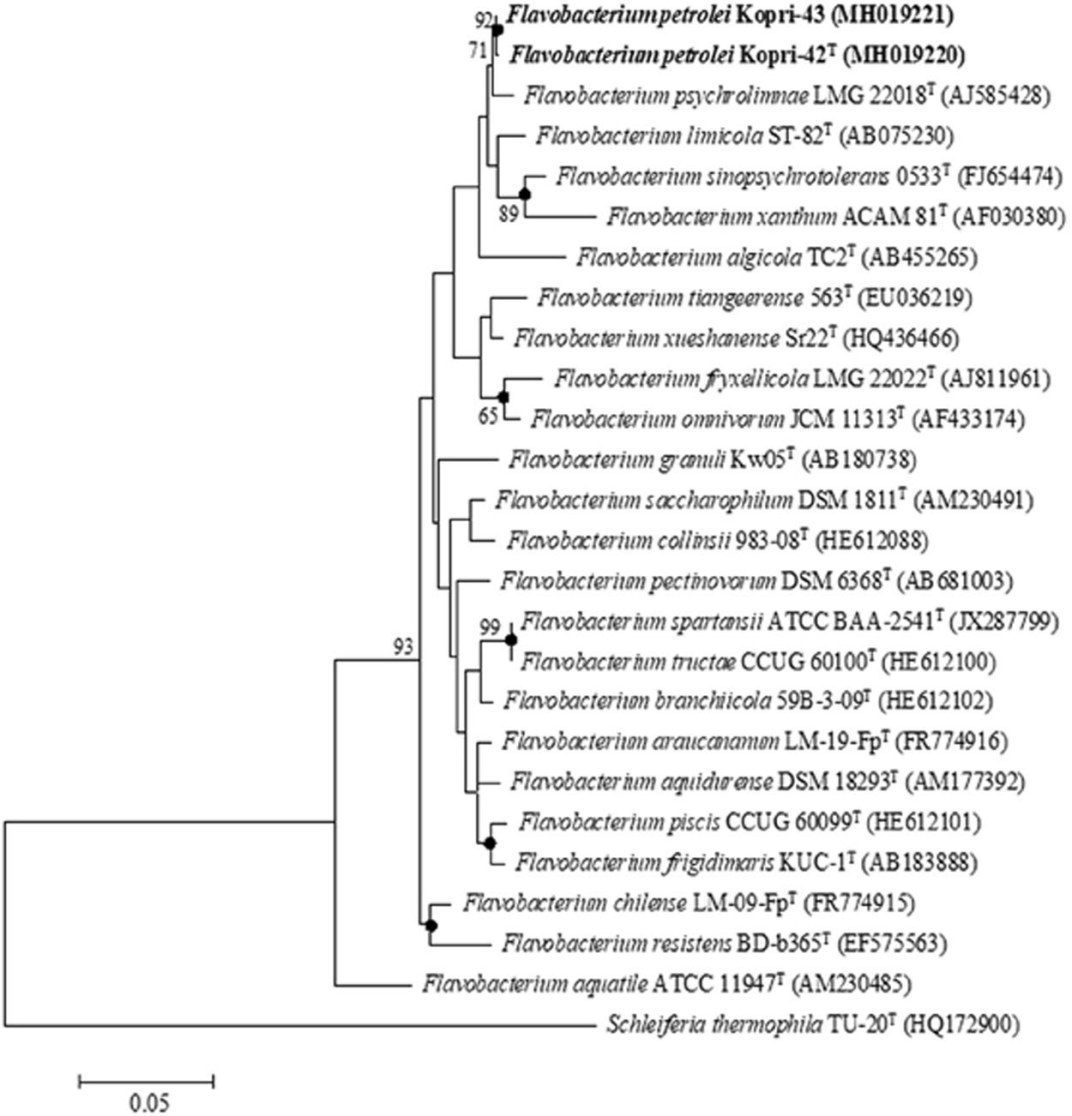
Phylogenetic tree inferred with maximum likelihood algorithm based on 16S rRNA gene sequences showing the relationship between strains Kopri-42^T^, Kopri-43, and closely related members of the genus *Flavobacterium*. Filled circles indicate branch-nodes recovered by maximum-likelihood, neighbor-joining, and maximum-parsimony phylogenetic trees. The numbers at the branch-nodes represent the percentage of 1,000 bootstrap replicates; only values >50% are depicted in the tree. GenBank accession numbers for 16S rRNA gene sequences are presented in parentheses. *Schleiferia thermophila* TU-20^T^ was included as an out-group. The scale bar represents 0.05 substitutions per nucleotide position.

The nearly complete length of 16S rRNA gene sequences of the strains Kopri-42^T^ and Kopri-43 were 1,444 and 1,438 bps, respectively. The comparative analysis of 16S rRNA gene sequence using the EZBioCloud server revealed that the strains Kopri-42^T^ and Kopri-43 belong to the genus *Flavobacterium* and shared highest sequence similarity with *F*. *psychrolimnae* LMG 22018^T^ (98.9% and 99.3%, respectively). The pairwise 16S rRNA gene sequence identity between strains Kopri-42^T^ and Kopri-43 was found to be 99.7%. The high 16S rRNA gene sequence similarity between strains Kopri-42^T^ and Kopri-43 revealed that both the strains could be considered as a single novel species. In addition, DNA-DNA hybridization (DDH) values between strain Kopri-42^T^ and Kopri-43 was 88.6 ± 2.1% (reciprocal, 83.9 ± 1.5%), indicating that Kopri-42^T^ and Kopri-43 represents two strains of the same genomospecies^16^. In addition, the DNA fingerprinting obtained by REP-PCR showed several similar bands between Kopri-42^T^ and Kopri-43 but differ with reference strains (see Fig. S1 in the supplementary material), suggesting that these two strains represent the same species. Based on the 16S rRNA gene sequence similarity, the other closest neighbors of the type strain Kopri-42^T^ were *F*. *limicola* DSM 15094^T^ (98.3%, sequence similarity); *F*. *tiangeerense* 0563^T^ (97.8%, sequence similarity); and *F*. *sinopsychrotolerans* 0533^T^ (97.7%, sequence similarity). The 16S rRNA gene sequence identities between the type strain Kopri-42^T^ and the closest phylogenetic relatives were in the range of 98.9–97.7%, which were below the threshold value of 98.7–99.0% used for species demarcation of prokaryotes^17,18^. These 16S rRNA gene sequence similarities values along with average nucleotide identity and *in silico* DNA-DNA hybridization (described below) suggested to allocate strain Kopri-42^T^ as a new species of the genus *Flavobacterium*. Furthermore, the phylogenetic trees analyses obtained from 16S rRNA gene sequence alignments by maximum-likelihood (Fig. 1); neighbor-joining (Fig. S2); and maximum-parsimony (Fig. S3) also showed that strains Kopri-42^T^ and Kopri-43 were grouped within the genus *Flavobacterium* and formed a cluster which consists cold-adaptive *Flavobacterium* species.

The whole genome sequence of strain Kopri-42^T^ contains 3738413 bp (GenBank accession number: QNVY00000000). The full genome was assembled in 22 contigs with an N50 of 693296 bp and genome coverage of 609.5x. The genome-based similarity calculated based on OrthoANIu between strain Kopri-42^T^ and closest reference strain *F*. *psychrolimnae* LMG 22018^T^ was 92.4%. The average nucleotide identity (ANI) values with other reference strains were below 90.0% (Table 1). These values were below the threshold ANI value of 95.0–96.0% used for delineating prokaryotic species^19^, suggesting strain Kopri-42^T^ is a novel strain of the genus *Flavobacterium*. In addition, *in silico* DNA-DNA hybridization (DDH) value between strain Kopri-42^T^ and *F*. *psychrolimnae* LMG 22018^T^ was 47.9%. The *in silico* DDH values with other reference strains were below 40.0% (Table 2). These values were significantly below the cut-off value of 70%, which clearly indicated that strain Kopri-42^T^ differs genetically from other type strains of genus *Flavobacterium* at the species level^16,20^. The G + C content of the chromosomal DNA for strains Kopri-42^T^ and Kopri-43 were 34.8 and 35.1 mol %, respectively, which is in agreement with the range of 32.0–38.0 mol % for the genus *Flavobacterim*^7,13^.

**Table 1:** The average nucleotide identity (ANI) and *in silico* DNA-DNA hybridization (DDH) values between strain Kopri-42^T^ and closely related reference strains.

Kopri-42^T^ vs.	ANI value (%)	*in silico* DDH (%)
*F*. *psychrolimnae* LMG 22018^T^	92.4	47.9
*F*. *limicola* DSM 15094^T^	89.8	39.1
*F*. *sinopsychrotolerans* 0533^T^	89.6	37.7
*F*. *xueshanense* sr22^T^	86.4	31.6
*F*. *tiangeerense* 0563^T^	78.8	22.2
*F*. *piscis* CCUG 60099^T^	75.8	21.1

**Table 2:** Differentiating characteristics of strain Kopri-42^T^, Kopri-43, and related species of the genus *Flavobacterium*.

Characteristic	1	2	3	4	5	6
Growth at 0 °C/30 °C	+/−	+/−	−/+	+/−	−/−	−/−
Highest salt tolerance (%, w/v)	1.0	1.0	1.5	1.5	1.5	0.5
pH range	6.0–10.5	6.0–10.5	6.5–11.0	5.5–10.5	6.5–10.0	6.5–9.5
Catalase/oxidase	+/−	+/−	+/+	+/+	+/+	w/+
**Hydrolysis of**						
Casein	−	−	+	+	+	+
Tween 40/60/80	−/−/−	−/−/−	+/+/−	w/−/−	−/−/−	+/w/−
Starch	−	−	−	−	−	+
DNA	−	−	+	−	+	−
**Enzyme activity**						
Esterase (C4)	w	w	w	+	+	w
Lipase (C 14)	−	−	w	w	w	w
Leucine arylamidase	+	w	+	+	+	w
Cystine arylamidase	w	+	+	+	w	+
Trypsin	w	w	w	w	w	−
*α*-chymotrypsin	w	w	w	w	+	w
Acid phosphatase	+	+	+	+	w	w
Naphthol-AS-BI-phosphohydrolase	−	−	w	w	−	−
*α*–glucosiadse	+	+	w	+	w	w
*β* –glucosiadse	+	+	w	−	−	−
*N*-acetyl-*β*-glucosaminidase	+	+	+	+	+	−
**Assimilation from**						
(API 20NE and API ID 32 GN)						
d-Mannose	+	+	+	−	−	+
Glycogen	+	+	+	+	+	−
Salicin	+	w	−	−	−	−
l-Proline	+	+	−	w	+	−
DNA G + C content (mol %)	34.8 (34.2)^#^	35.1	33.8–34.5^a^	35.0^b^	32.5^c^	34.8^d^

Strains: 1, Kopri-42^T^; 2, Kopri-43; 3, *F*. *psychrolimnae* KACC 11737^T^; 4, *F*. *limicola* KACC 11965^T^; 5, *F*. *sinopsychrotolerans* JCM 16398^T^; 6, *F*. *tiangeerense* JCM 15087^T^. All data presented in the table are generated from this study, unless marked. ^a^Data from Trappen *et al*.^13^; ^b^Data from Tamaki *et al*.^5^; ^c^Data from Xu *et al*.^15^; ^d^Data from Xin *et al*.^14^; ^#^value determined from genome sequence. +, positive; −, negative; w, weakly positive.

**Figure 2:**
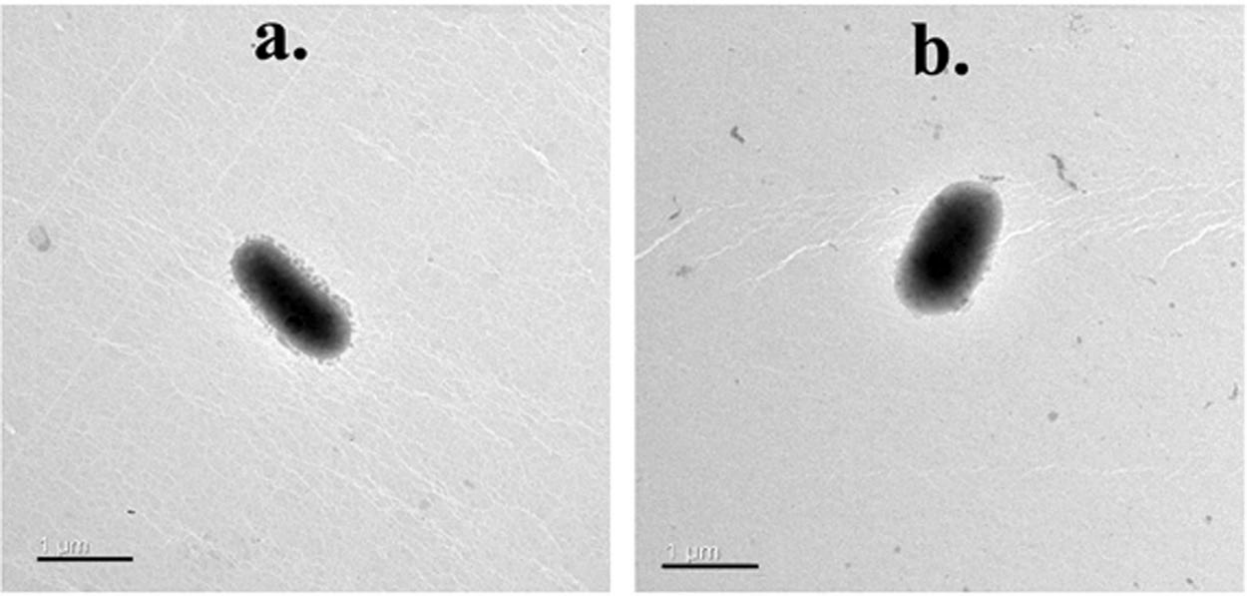
Transmission electron photomicrographs of the cells of novel strains grown on R2A agar at 10 °C for 5 days. (**a**) Kopri-42^T^; (**b**) Kopri-43. Bar: 1.0 µm.

Both strains were Gram-stain-negative, non-motile, and aerobic. The photomicrographs obtained from transmission electron microscopy revealed that cells of strains Kopri-42^T^ and Kopri-43 were rod-shaped without flagella (Fig. 2). The colonies of both strains on R2A agar were yellow, 1.0–2.0 mm in diameter, circular, convex, translucent, and glistening with entire margins. Strains Kopri-42^T^ and Kopri-43 grew well on R2A, TSA, and NA; poorly grew on LB, marine agar, and veal infusion agar; and did not grow on MacConkey agar. Growth was detected at temperatures 0–25 °C, pH 6.0–10.5, and 0–1% (w/v) NaCl concentration (Table 2). The optimum temperature for strain Kopri-42^T^ was 10–15 °C as revealed from the growth curve (Fig. S4). Based on the growth temperature data, both strains are concluded to be psychrophilic bacteria. The presence of higher amount of cellular unsaturated fatty acids may contribute to the cold adaptation of these strains (Table 3). It has been documented that presence of higher proportion of unsaturated fatty acids content in the cell membrane favors bacteria to grow at low temperature^21,22^. Both strains were positive for catalase test but negative for oxidase test. However, oxidase test was positive for all the reference strains considered in this study. Production of flexirubin-type pigment was absent from both strains. Hydrolyze aesculin and CM-cellulose but cannot hydrolyze Tween (40/60/80), starch, casein, DNA, and tyrosine. Strain Kopri-42^T^ was sensitive to novobiocin, tetracycline, nalidixic acid, rifampicin, cyclohexamide, trimethoprim, sulfamethoxazole and chloramphenicol but resistant to kanamycin, neomycin, gentamycin, penicillin, ampicillin, and streptomycin. Other differentiating properties and phenotypic traits resulted from API ZYM, API 20NE, and API ID 32GN test kits of strains Kopri-42^T^ and Kopri-43 are depicted in the digital protologue table and presented along with other nearest phylogenetic members (Tables 2 and S1).

**Table 3:** Cellular fatty acid profile of strain Kopri-42^T^, Kopri-43, and related species of the genus *Flavobacterium*.

Fatty acid	1	2	3	4	5	6
**Saturated**						
C_14:0_	0.5	0.6	1.0	0.5	0.8	0.6
C_16:0_	1.6	1.8	0.8	2.8	0.9	1.6
iso-C_15:1_ G	5.8	5.3	9.2	2.6	8.3	3.6
iso-C_16:1_ H	5.4	5.1	2.3	9.3	5.1	6.6
anteiso-C_13:0_	—	—	1.5	—	—	—
anteiso-C_15:1_ A	1.0	0.9	2.5	0.8	0.6	1.3
anteiso-C_15:0_	8.0	9.3	19.0	7.4	4.9	9.1
iso-C_13:0_	—	—	1.3	—	—	—
iso-C_14:0_	3.0	2.8	2.2	4.3	4.7	4.0
iso-C_15:0_	9.8	10.1	15.4	4.3	11.8	4.8
iso-C_16:0_	5.7	5.2	4.6	11.8	4.6	6.9
iso-C_17:0_	0.7	0.6	—	—	—	—
**Unsaturated**						
C_15:1_ *ω*6*c*	11.6	11.5	1.3	5.4	14.5	11.1
C_16:1_ *ω*5*c*	0.3	0.3	—	0.3	0.5	0.4
C_17:1_ *ω*6*c*	6.1	5.8	0.6	3.5	5.9	7.4
C_17:1_ *ω*8*c*	1.0	1.0	0.2	0.5	0.5	1.2
anteiso-C_17:1_ *ω*9*c*	—	—	3.4	1.1	—	—
C_18:1_ *ω*5*c*	—	—	1.3	0.6	—	0.6
**Hydroxy**						
iso-C_14:0_ 3-OH	0.4	0.3	0.5	0.9	0.5	0.9
C_15:0_ 2-OH	0.4	0.4	1.9	0.7	0.3	0.7
C_15:0_ 3-OH	1.7	1.7	—	—	1.9	1.5
iso-C_15:0_ 3-OH	4.7	4.5	6.1	2.9	6.8	3.9
C_16:0_ 3-OH	0.8	0.7	2.4	1.3	1.2	1.3
iso-C_16:0_ 3-OH	9.2	9.7	2.8	12.6	7.8	10.7
C_17:0_ 2-OH	0.9	1.0	2.1	0.8	0.3	0.7
C_17:0_ 3-OH	0.4	0.3	0.2	—	0.2	0.5
iso-C_17:0_ 3-OH	5.3	5.9	4.3	2.9	3.6	3.5
Summed features*						
3	10.1	10.3	8.7	19.0	10.2	12.8
9	4.4	4.0	2.5	2.1	3.1	2.4

Strains: 1, Kopri-42^T^; 2, Kopri-43; 3, *F*. *psychrolimnae* KACC 11737^T^; 4, *F*. *limicola* KACC 11965^T^; 5, *F*. *sinopsychrotolerans* JCM 16398^T^; 6, *F*. *tiangeerense* JCM 15087^T^. All data presented below in the table are generated from the present study. Values are percentages of total fatty acids. Values with <0.2% of the total fatty acids are not presented; –, not detected or <0.2%.

*Summed features represent groups of two or three fatty acids that could not be separated using the MIDI system. Summed feature 3 contained C_16:1_*ω*7*c* and/or C_16:1_*ω*6*c*, Summed feature 9 contained iso-C_17:1_*ω*9*c* and/or C_16:0_10-methyl.

Both strains comprise menaquinone-6 (MK-6) (Fig. S5) as sole respiratory quinone which is typical to menaquinone system of the genus *Flavobacterium*^8^. Strains Kopri-42^T^ and Kopri-43 contain phosphatidylethanolamine (PE) as the major polar lipid. The unidentified aminolipids (AL1-AL6) and unidentified lipids (L1–L4) were also detected in minor amount (Fig. S6a,b). The major polar lipid profile of both strains shared similar pattern with *F*. *psychrolimnae* KACC 11737^T^ (Fig. S6c). However, some proportional difference exists in the minor polar lipid profile between strains Kopri-42^T^, Kopri-43 and *F*. *psychrolimnae* KACC 11737^T^ (Fig. S6). The major cellular fatty acids present in Kopri-42^T^ were C_15:1_*ω*6*c* (11.6%), summed feature 3 (C_16:1_*ω*7*c* and/or C_16:1_*ω*6*c*; 10.1%), iso-C_15:0_ (9.8%), iso-C_16:0_ 3-OH (9.2%), anteiso-C_15:0_ (8.0%), iso-C_15:1_ G (5.8%), iso-C_16:1_ H (5.4%), and iso-C_17:0_ 3-OH (5.3%). The observed patterns of fatty acids are similar to closest neighbors. Despite of the comprehensive similarities, a differential pattern exhibited with minor amount of fatty acids. Both strains detected iso-C_17:0_ which was absent from closest members. C_15:0_ 3-OH was present in strains Kopri-42^T^ and Kopri-43 but not detected from *F*. *psychrolimnae* KACC 11737^T^ and *F*. *limicola* KACC 11965^T^. Most of the reference strains had anteiso-C_17:1_*ω*9*c* and C_18:1_*ω*5*c* but absent from strains Kopri-42^T^ and Kopri-43 (Table 3).

**Figure 3:**
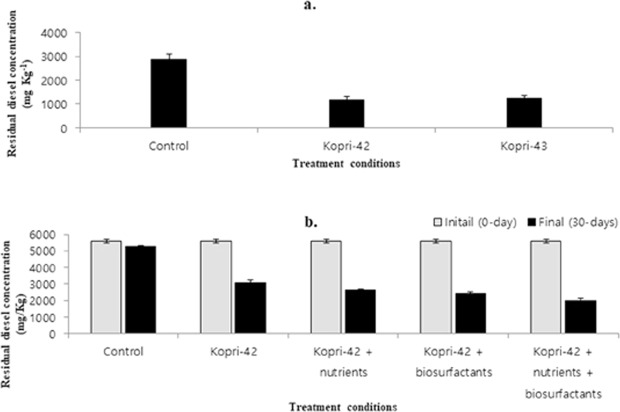
Changes in diesel concentration in different treatment conditions. (**a**) in MSM liquid, treated for 14 days; and (**b**) in the standard soil, treated for 30-days. Bar represents the standard error of the duplicate samples.

Both the strains degraded substantial amount of diesel in liquid media and soil environment. In MSM liquid media, strains Kopri-42^T^ and Kopri-43 degraded 60.0% and 58.3% of diesel oil, respectively (Fig. 3a). Statistical analysis indicates that the rates of diesel degradation by strains Kopri-42^T^ and Kopri-43 were not significantly different (*p* > 0.05). In diesel contaminated soil, the degradation rate was improved with various treatment conditions. When the diesel contaminated soil was treated with only bacterial strain Kopri-42^T^, the remediation efficiency was 44.8%. The degradation rate was found to be enhanced (64.8%) when the contaminated soil was treated with Kopri-42^T^ + nutrients + biosurfactants (Figs 3b and S7). Statistical analysis showed that the rates of diesel degradation between various treatment conditions were significantly different (*p* < 0.05). This result indicates that strain Kopri-42^T^ and Kopri-43 can be used as biological agent for remediating diesel oil from cold environments.

In conclusion, 16S rRNA gene sequence analysis, phylogenetic trees, and chemotaxonomic data indicate both strains clearly belong to the genus *Flavobacterium*. The differentiating phenotypic properties and chemotaxonomic data presented in Tables 2 and 3 preliminary distinguish the two strains as members of a new species in the genus *Flavobacterium*. The genome sequence characteristics, OrthoANIu and DDH values, and REP-PCR confirm that both strains represent single novel species. Furthermore, both psychrophilic strains can degrade diesel oil and able to thrive in oil-contaminated cold environments indicating their significance in the bioremediation field. Considering the above mentioned genotypic, phylogenetic, phenotypic, biochemical, and chemotaxonomic data, both strains are concluded to represent a novel species in the genus *Flavobacterium*, with the proposed name *Flavobacterium petrolei* sp. nov. The type strain is Kopri-42^T^ (=KEMB 9005-710^T^ = KACC 19625^T^ = NBRC 113374^T^). The formal proposal of the new species name *Flavobacterium petrolei* sp. nov. is given in the Table S1 with the TaxonNumber TA00628 (http://imedea.uib-csic.es/dprotologue/edit_entryForm.php?form_id=10891&entry_id=628).

## Materials and Methods

### Bacterial isolation, growth conditions, and maintenance of strain

For isolation, soil samples were collected during August and september 2012 from different corners of the base station near the DASAN, Korean Arctic Station, N-9173 Ny-Alesund, Norway (GPS location: 78°55′30.13″N 11°55′20.21″E). The diesel oil used in this study was purchased from petroleum station (GS Caltex, Suwon, South Korea). The composition of mineral salt medium (MSM) used for isolation of oil-degrading bacteria is given in Table S2. An enrichment cultivation technique employing Transwell plate (Corning) was implemented for the isolation of psychrophilic oil-degrading bacteria as described previously^9,23^. Pure isolates were obtained after multiple streaking bacterial colonies on R2A (MB Cell) agar incubating at 10 °C. All the strains isolated during this study are listed in Table S3. The pure culture of strains Kopri-42^T^ and Kopri-43 grown in R2A agar was temporarily stored at 4 °C and subcultured regularly at the interval of two weeks until the taxonomic study was completed. For long-term storage both strains were stored at −80 °C in R2A broth with 20% (v/v) glycerol and later deposited permanently in culture collection center.

### Genotypic characterization and phylogenetic analysis

Two strains Kopri-42^T^ (=KEMB 9005-710^T^ = KACC 19625^T^ = NBRC 113374^T^) and Kopri-43 (=KEMB 9005-7101 = KACC 19626 = NBRC 113411) isolated from Arctic soil were characterized in this study. Genomic DNA from strains Kopri-42^T^ and Kopri-43 was isolated using a DNA isolation kit following the exact protocol provided with the kit (InstaGene Matrix kit, Bio-Rad, USA). The conditions of PCR amplification, primers information, purification of PCR products, and sequencing and analysis of 16S rRNA gene were performed following a previously described protocol^19,24^. For DNA fingerprinting, repetitive extragenic palindromic (REP) PCR was performed using BOXA1R primer (5′- CTACGGCAAGGCGACGCTGACG-3′) following the conditions as mentioned previously^25^. The gel-electrophoresis image of PCR product was analyzed and the dendrogram was generated using PyElph 1.4 software by UPGMA method, with a bootstrap of 100. The similarity matrix was computed using Dice coefficient^26^.

The nearest phylogenetic members were determined by comparing 16S rRNA gene sequences of strains Kopri-42^T^ and Kopri-43 with the sequences available in the GenBank database (https://blast.ncbi.nlm.nih.gov/Blast.cgi) and EZBioCloud server^27^. For phylogenetic analysis, multiple alignment with the sequences of closest neighbors was carried out using CLUSTAL X 2.1^28^. Gaps between the 5′ and 3′ ends of the aligned sequences were deleted utilizing the BioEdit program^29^. Phylogenetic trees were inferred with the help of software package MEGA6^30^ by using three different algorithms: the neighbor-joining^31^, maximum-parsimony^32^ and maximum-likelihood^33^. Kimura two-parameter model^32^ was used to calculate evolutionary distances and the bootstrap values were calculated based on 1000 replicates^34,35^.

For whole genome sequencing, genomic DNA from strain Kopri-42^T^ was extracted using the commercial kit (Genomic DNA Purification Kit, Wizard, Promega) following the manufacturer’s protocol. The whole genome sequence of strain Kopri-42^T^ was obtained using an Illumina MiSeq sequencer and assembled utilizing an assembly toolkit software SPAdes v3.10.1 at the ChunLab (Seoul, South Korea). The genome sequence was annotated using the NCBI Prokaryotic Genome Annotation Pipeline (PGAP, 2013). The genomic relatedness of the novel strain Kopri-42^T^ with the whole genome shotgun sequences of closely related species of *Flavobacterium* was determined based on the Average Nucleotide Identity using USEARCH software tool (OrthoANIu)^36^. *In silico* DDH was calculated using Genome-to-GenomeDistance Calculator (GGDC 2.1; http://ggdc.dsmz.de/ggdc.php) with recommended BLAST + alignment and formula 2 (identities/HSP length)^20^.

The G + C content of the chromosomal DNA for strains Kopri-42^T^ and Kopri-43 was determined by real-time PCR analysis^37^ and also genomic G + C content for the type strain was calculated based on the whole genome sequence data. DNA-DNA hybridization (DDH) was conducted between strain Kopri-42^T^ and Kopri-43 as described previously^38^. Salmon sperm was considered as the negative control and photobiotin was used as probes to label genomic DNA of strain Kopri-42^T^. The values of DDH were determined fluorometrically in microplate wells using a 1420 Multilabel Counter (Perkin Elmer). Additionally, for reverse hybridization DNA of strain Kopri-43 was labeled with photobiotin and used as a probe to strain Kopri-42^T^. All the experiments were performed in triplicate.

### Selection of reference strains

Based on preliminary 16S rRNA gene sequence and the phylogenetic analysis, *Flavobacterium psychrolimnae* KACC 11737^T^, *Flavobacterium limicola* KACC 11965^T^, *Flavobacterium sinopsychrotolerans* JCM 16398^T^, and *Flavobacterium tiangeerense* JCM 15087^T^ were considered for comparative morphological, physiological, biochemical, and chemotaxonomic studies. In addition to the above type strains, *Flavobacterium piscis* CCUG 60099^T^ and *Flavobacterium xueshanense* sr22^T^ were also selected for genomic relatedness (ANI and *in silico* DDH) study.

### Morphological, physiological and biochemical characterization

Cellular morphologies of the strains Kopri-42^T^ and Kopri-43 cultivated on R2A for 5 days at 10 °C were observed by transmission electron microscopy (Libra 120 EFTEM; Zeiss). Colony morphologies of both strains on R2A agar were determined using a Zoom Stereo Microscope (SZ61; Olympus Japan) after incubation at 10 °C for 5 days on TSA. Gram staining type was studied following the protocol described by Doetsch^39^. Cellular motility, growth ability on various commercial culture media, temperature range, pH range, NaCl tolerance, catalase, and oxidase tests, spore staining, and DNA degradation assay were determined as illustrated previously^9,40^. To determine psychrophilic optimum temperature, the growth curve was determined at 4, 10, 15, 20, and 25 °C by measuring growth absorbance at 600 nm using visible spectrophotometer (Biochrome Libra S4). Anaerobic growth was assessed after 14 days incubation at 10 °C using an anaerobic jar (BBL, Becton Dickinson) with Gas Generating Pouch System (GasPak^TM^ EZ). Indole test and H_2_S production were evaluated in SIM medium. MR-VP broth was used to perform the MR-VP test. Hydrolysis of Tween 40, Tween 60, Tween 80, starch, gelatin, aesculin, casein, tyrosine, and CM-cellulose was performed as previously described^9,41^. The presence of flexirubin-type pigments was analyzed with 20% (w/v) KOH solution^42^. Antibiotic susceptibility test was performed on R2A plates by the paper disc method^43^ with the following commercial antibiotics: tetracycline (30 µg), kanamycin (30 µg), rifampicin (10 µg), nalidixic acid (30 µg), novobiocin (30 µg), neomycin (30 µg), streptomycin (10 µg),gentamycin (10 µg), ampicillin (30 µg), penicillin (10 µg), chloramphenicol (100 µg), cyclohexamide (30 µg), trimethoprim (30 µg), and sulfamethoxazole (30 µg). API ZYM, API 20NE, and API ID 32GN test kits (bioMérieux) were used to determine other enzymatic, physiological, biochemical, and assimilation properties following the manufacturer’s instructions.

### Chemotaxonomic characterization

The respiratory quinone and polar lipids were extracted and analyzed from freeze-dried cells following the method as presented previously^44,45^. The TLC chromatograms of polar lipids were sprayed with appropriate detection reagents for visualization of various spots as described previously^9,45,46^. The cellular fatty acids of strains Kopri-42^T^, Kopri-43, and reference strains were extracted after late log phase grown at 10 °C on R2A using the standard MIDI protocol (Sherlock Microbial Identification System, version 6.0B) and analyzed with a gas chromatograph (6890 Series GC System; Hewlett Packard) using the TSBA6 database of the Microbial Identification System^47^.

### Determination of diesel degradation ability

The preliminary diesel degrading ability of strains Kopri-42^T^ and Kopri-43 was assessed in MSM liquid media containing 3000 ppm diesel oil at 10 °C as described previously^23^. Furthermore, the degrading ability was analyzed in standard soil contaminated with diesel oil. The diesel-contaminated soil was prepared in the lab using sand, kaolin, peat moss, and the diesel oil at 600 mg kg^−1^. All the components were mixed thoroughly and stabilized for 1 month prior to conducting the experiment. The physio-chemical parameters of the prepared soil were studied after stabilization and have been presented in Table S4. The diesel degradation experiment was performed in the plastic vessel (size: 24 cm × 17 cm × 16 cm) each containing 2.5 Kg of prepared soil. Diesel-remediation was evaluated in five different treatment conditions (Table S5). The bacterial inoculums, nutrients, and biosurfactants were delivered to the experimental vessels through solution-pouring technique at the 5-day interval for 1 month. The nutrients in the respective treatment conditions were added maintaining the carbon, nitrogen, and phosphorous at the ratio of 100:10:1. All the experimental vessels were incubated at 10 °C in an incubator. After 1-month treatment, residual diesel concentration was determined using GC-FID (HP 6890, Agilent, USA) following the procedure as explained previously^48^.

The data of diesel degradation were subjected for statistical analysis to determine mean, standard deviation (SD), and standard error. The *p*- value between different treatments were calculated using two-way ANOVA in the Microsoft Office Excel 2013. The *p*- value less than 0.05 was used to conclude significant differences between various treatment conditions.

### Accession numbers

The GenBank/EMBL/DDBJ accession numbers for the 16S rRNA gene sequence of strains Kopri-42^T^ and Kopri-43 are MH019220 and MH019221, respectively. This Whole Genome Shotgun project has been deposited at DDBJ/ENA/GenBank under the accession QNVY00000000. The version described in this paper is version QNVY02000000.

## Supplementary information

Additional Tables and Figures
