# Supplementary material for: Flavobacterium petrolei sp. nov., a novel psychrophilic, diesel-degrading bacterium isolated from oil-contaminated Arctic soil
Source: Sci Rep. 2019 Mar 11;9:4134. doi: 10.1038/s41598-019-40667-7 (PMC6411956; doi:10.1038/s41598-019-40667-7)
Supplement: Supplementary file 1 — Additional Tables and Figures [file 41598_2019_40667_MOESM1_ESM.pdf]

Supplementary Materials

Scientific Reports

Flavobacterium petrolei sp. nov., a novel psychrophilic, diesel-degrading bacterium isolated from oil-contaminated Arctic soil

Dhiraj Kumar Chaudhary[a], Dong-Uk Kim[b], Dockyu Kim[c] and Jaisoo Kim[a] *

[a]Department of Life Science, College of Natural Sciences, Kyonggi University, Suwon, Gyeonggi-Do 16227, South Korea

[b]Division of Bio-convergence, College of Convergence and Integrated Science, Kyonggi University, Suwon, Gyeonggi-Do 16227, South Korea

[c]Division of Polar Life Sciences, Polar Research Institute, Incheon 21990, South Korea

Running title: *Flavobacterium petrolei* sp. nov.

Contents category: Systematic-New Taxa; Subsection: *Bacteroidetes*

*Corresponding author: Jaisoo Kim.

Tel: +82-31-249-9648; Fax: +82-31-253-1165; E-mail: jkimtamu@kgu.ac.kr

Table S1. Species description of *Flavobacterium petrolei* sp. nov. according to the Digital Protologue database TaxonNumber TA00628.

**Taxonumber**	TA00628
**Species name**	*Flavobacterium petrolei*
**Genus name**	*Flavobacterium*
**Specific epithet**	*petrolei*
**Species status**	sp. nov.
**Species etymology**	pe.tro'le.i. N.L. gen. n. *petrolei*, of petroleum
**Designation of the type strain**	Kopri-42
**Strain collection numbers**	KEMB 9005-710^{T}= KACC 19625^{T}= NBRC 113374^{T}
**16S rRNA gene accession number**	MH019220
**Genome accession number**	QNVY00000000
**Genome status**	Complete
**Genome size**	3861
**GC mol%**	34.0
**Country of origin**	Norway
**Region of origin**	The DASAN, Korean Arctic Station, N-9173 Ny-Alesund
**Date of isolation**	2018/1/10
**Source of isolation**	Arctic soil contaminated by petroleum oil
**Sampling date**	2018/8/15
**Geographic location**	The DASAN, Korean Arctic Station, N-9173 Ny-Alesund
**Latitude**	78°55′30.13"N
**Longitude**	11°55′20.21"E
**Number of strains in study**	2
**Source of isolation of non-type strains**	Arctic soil contaminated by petroleum oil
**Growth medium, incubation conditions[temperature, pH, and further information] used for standard cultivation**	R2A medium at 10°C
**Alternative medium 1**	Tryptone soya agar (TSA)
**Alternative medium 2**	Nutrient agar (NA)
**Conditions of preservation**	In R2A broth supplemented with 20 % (v/v) glycerol and stored at -80 °C<br>or by lyophilization with 20 % (w/v) skimmed milk.
**Gram stain**	Negative
**Cell shape**	Rod
**Cell size (length or diameter)**	1.2-1.5 µm long and 0.6-0.7 µm wide
**Motility**	Non-motile
**Colony morphology**	Colonies on R2A are yellow with size of 1.0–2.0 mm in diameter, circuar, convex, translucent, and glistening with entire margins.
**Temperature optimum**	10-15
**pH optimum**	6.5-8.5
**pH category**	Neutrophile
**Salinity optimum**	0
**Salinity category**	Neutrophile
**Relationship to O_2**	Aerobe
**O_2 conditions for strain testing**	Aerobiosis
**Positive tests with API**	**API ZYM:** Alkaline phosphatase, esterase (C4; weak), esterase lipase (C8), leucine arylamidase, valine arylamidase, cystine arylamidase (weak), trypsin

	(weak), α-chymotrypsin (waek), acid phosphatase, α-glucosidase, β-glucosidase, and *N*-acetyl-β-glucosaminidase<br><br>**API 20 NE:** ESC, PNPG, GLU, MNE, and MAL<br><br>**API ID 32 GN:** SAC, MAL, GLYG, GLU, SAL, and PRO
**Negative tests with API**	**API ZYM:** Lipase (C14), naphthol-AS-BI-phosphohydrolase, α-galactosidase, β-galactosidase, β-glucuronidase, α-mannosidase, and α-fucosidase<br><br>**API 20 NE:** NO_3, TRP, GLU (fermentation), ADH, URE, GEL, ARA, MAN, NAG, GNT, CAP, ADI, MLT, CIT, and PAC<br><br>**API ID 32 GN:** RHA, NAG, RIB, INO, ITA, SUB, MNT, ACE, LAT, ALA, 5KG, mOBE, SER, MAN, MEL, FUC, SOR, ARA, PROP, CAP, VALT, CIT, HIS, 2KG, 3OBU, and pOBE
**Commercial kits used**	API ZYM, API 20 NE, API ID 32 GN
**Energy metabolism**	Chemoorganotroph
**Oxidase**	Negative
**Catalase**	Positive
**Positive tests**	Hydrolysis of Aesculin and CM-cellulose
**Negative tests**	Production of H2S, indole production, methyl red test, Voges-Proskauer test, Flexirubin-type pigment production, and hydrolysis of casein, Tween (40, 60, 80), starch, gelatin, DNA, and tyrosine
**Major fatty acids**	$C_{15:1}\omega 6c$, summed feature 3 ($C_{16:1}\omega 7c$ and/or $C_{16:1}\omega 6c$), iso-$C_{15:0}$, iso-$C_{16:0}$ 3-OH, anteiso-$C_{15:0}$, iso-$C_{15:1}$ G, iso-$C_{16:1}$ H, and iso-$C_{17:0}$ 3-OH
**Biosafety level**	1
**Habitat**	Oil-contaminated soil (ENVO: 00002875)
**Biotic relationship**	Free-living
**Known pathogenicity**	None

Table S2. Composition of mineral salt media used for isolation of oil-degrading bacteria in this study.

Composition	Amount (g L^{-1})
KH_2PO_4	1.8
K_2HPO_4	1.8
KNO_3	0.9
$(NH_4)_2SO_4$	1.5
NaCl	0.5
$MgSO_4 \cdot 7H_2O$	0.4
$CaCl_2 \cdot 2H_2O$	0.02
$FeCl_3 \cdot 7H_2O$	0.01
Trace element solution*	1 mL
Vitamin solution#	1 mL
Distilled water (d/w)	1000 mL
Diesel oil	5 mL

*The composition of trace element solution (g L^{-1}): $ZnSO_4 \cdot 7H_2O$, 0.1; $MnCl_2 \cdot 4H_2O$, 0.03; H_2BO_3, 0.3; $CoCl_2 \cdot 6H_2O$, 0.2; $CuCl_2 \cdot 2H_2O$, 0.01; $NiCl_2 \cdot 6H_2O$, 0.02; $Na_2MoO_4 \cdot 2H_2O$, 0.03.

#The composition of vitamin solution (g L^{-1}): Biotin, 0.01; nicotinamide, 0.035; thiamine dichloride, 0.03; p-aminobenzoic acid,0.02; pyridoxal chloride, 0.01; Ca-pantothenate, 0.01; and vitamin B12, 0.005

Trace element, vitamin solution, and diesel oil were filtered sterilized and then added in the media after autoclave.

pH= 7.2± 0.2 at 25 ºC.

Table S3. Arctic strains isolated from Arctic soil samples during the study of psychrophilic oil-degrading bacteria. The strains depicted in bold were subjected for taxonomic investigation in this study. The soil samples were collected during August and September 2012. The sampling sites were different corners of the base station near The DASAN, Korean Arctic station, N-9173 Ny-Alesund, Norway (GPS location: 78°55′30.13"N 11°55′20.21"E). All the strains have been deposited in the GenBank and the accession no. is given in the table below.

**Strains**	**Closest species**	**Similarity (%)**	**GenBank accession no.**
KOPRI-1	*Flavobacterium sinopsychrotolerans* 0533^{T}	99.93	MH018886
KOPRI-2	*Pseudomonas arsenicoxydans* CECT 7543^{T}	99.93	MH018887
KOPRI-4	*Rhodococcus quingshengii* JCM 15477^{T}	100.00	MH018888
P-39-1	*Pseudomonas marginalis* ATCC 10844^{T}	98.67	MH018919
P-38-1	*Pseudomonas prosekii* LMG 26867^{T}	99.11	MH018918
P-36-1	*Paeniglutamicibacter sulfurous* DSM 20167^{T}	99.17	MH018917
P-35-1	*Paeniglutamicibacter antarcticus* SPC26^{T}	98.90	MH018916
P-34-3	*Paeniglutamicibacter psychrophenolicus* AG31^{T}	99.24	MH018915
P-34-2	*Arthrobacter citreus* DSM 20133^{T}	99.45	MH018914
P-33-1	*Carnobacterium viridians* MPL-11^{T}	99.53	MH018913
P-32-1	*Arthrobacter psychrochitiniphilus* GP3^{T}	98.84	MH018912
P-30-1	*Pseudarthrobacter siccitolerans* 4J27^{T}	99.24	MH018911
P-29-2	*Pseudarthrobacter oxydans* KCTC 3383^{T}	99.24	MH018910
P-29-1	*Pseudarthrobacter sulfonivorans* ALL^{T}	99.23	MH018909
P-25-2	*Carnobacterium viridians* MPL-11^{T}	98.72	MH019223
P-25-1	*Paenibacillus antarcticus* CECT 5836^{T}	99.39	MH018908
K-38	*Pseudomonas helmanticensis* OHA11^{T}	98.90	MH018885
Kopri-47	*Pseudomonas avellanae* BPIC 631^{T}	98.97	MH018892
Kopri-46	*Paeniglutamicibacter antarcticus* SPC26^{T}	98.90	MH019222
Kopri-35	*Arthrobacter alpines* DSM 22274^{T}	99.18	MH018890
Kopri-28	*Arthrobacter psychrochitiniphilus* GP3^{T}	99.25	MH018889
Kopri-49	*Janthinobacterium lividum* DSM 1522^{T}	99.59	MH018893
**Kopri-43**	***Flavobacterium psychrolimnae* LMG 22018^{T}**	**99.30**	**MH019221**
**Kopri-42**	***Flavobacterium psychrolimnae* LMG 22018^{T}**	**99.09**	**MH019220**
Kopri-41	*Pseudomonas migulae* CIP 105470^{T}	99.79	MH018891
K-13P	*Pseudomonas fluorescens* DSM 50090^{T}	99.86	MH018907

KP-12-3	*Pseudomonas brassicacearum subsp. Neoaurantica* ATCC 49054^{T}	99.45	MH018906
KP-10-2	*Pseudomonas veronii* DSM 11331^{T}	99.73	MH018904
KP-7-2	*Pseudomonas frederiksbergensis* JAJ28^{T}	98.70	MH018902
KP-4-3	*Pseudomoans arsenicoxydans* CECT 7543^{T}	99.66	MH018899
KP-2-2	*Caballeronia udeis* LMG 27134^{T}	99.45	MH018897
KP-1-4	*Pseudomonas yamanorum* 8H1^{T}	100.00	MH018895
KP-11-2	*Pseudomonas exremaustralis* 14-3^{T}	99.79	MH018905
KP-10-1	*Pseudomonas antarctica* CMS 35^{T}	99.93	MH018903
KP-7-1	*Pseudomoans frederiksbergensis* JAJ28^{T}	98.56	MH018901
KP-6-1	*Pseudomonas mandelii* CIP 105273^{T}	99.73	MH018900
KP-3-1	*Janthinobacterium svalbardensis* JA-1^{T}	99.79	MH018898
KP-2-1	*Pseudomonas caspiana* FBF 102^{T}	99.56	MH018896
KP-1-2	*Pseudomonas graminis* DSM 11363^{T}	99.79	MH018894
PLR-1-1	*Paenisporosarcina macmurdoensis* CMS 21w^{T}	99.73	MH018920
PLR-15-1	*Arthrobacter psychrochitiniphilus* GP3^{T}	99.31	MH018921
PLR-15-2	*Arthrobacter citreus* DSM 20133^{T}	99.24	MH018922
PLR-15-3	*Rahnella Victoriana* FRB 225^{T}	99.41	MH018923
PLR-18-2	*Arthrobacter citreus* DSM 20133^{T}	99.31	MH018925
PLR-15-4	*Paenibacillus macquariensis subsp. Macquariensis* ATCC 23464^{T}	99.93	MH018924

Table S4. Characteristics of diesel-contaminated soil.

Parameters	Values
Sand (0.020-2.000 mm)	80.0 %
Silt (0.002-0.020 mm)	10.0 %
Clay (<0.002 mm)	10.0 %
pH_{H20} (1:2)	6.5 ± 0.5
Total iron	1.0 %
Organic matter	5.1 %
Water content	1.2 %
Bulk density	1.1 g ml^{-1}
Porosity	57.2 %
Colony forming unit	1.1×10^{2} CFU g^{-1}
Diesel concentraiton	5600 mg Kg^{-1}
Soil texture	Loamy sand

Table S5. Experimental set-up for diesel-degradation in soil.

Experiment set-up	Treatment condition
Control (Ctrl)	Treated with water
Kopri-42	Treated with strain Kopri42^T (8 x 10^7 CFU g^{-1})
Kopri-42 + nutrients	Treated with strain Kopri42^T and nutrients (NH_4NO_3 + KH_2PO_4)
Kopri-42 + biosurfactants	Treated with strain Kopri42^T and alpha olefin sulfonate (0.1%)
Kopri-42 + nutrients + biosurfactants	Treated with strain Kopri42^T ; NH_4NO_3 + KH_2PO_4; and alpha olefin sulfonate

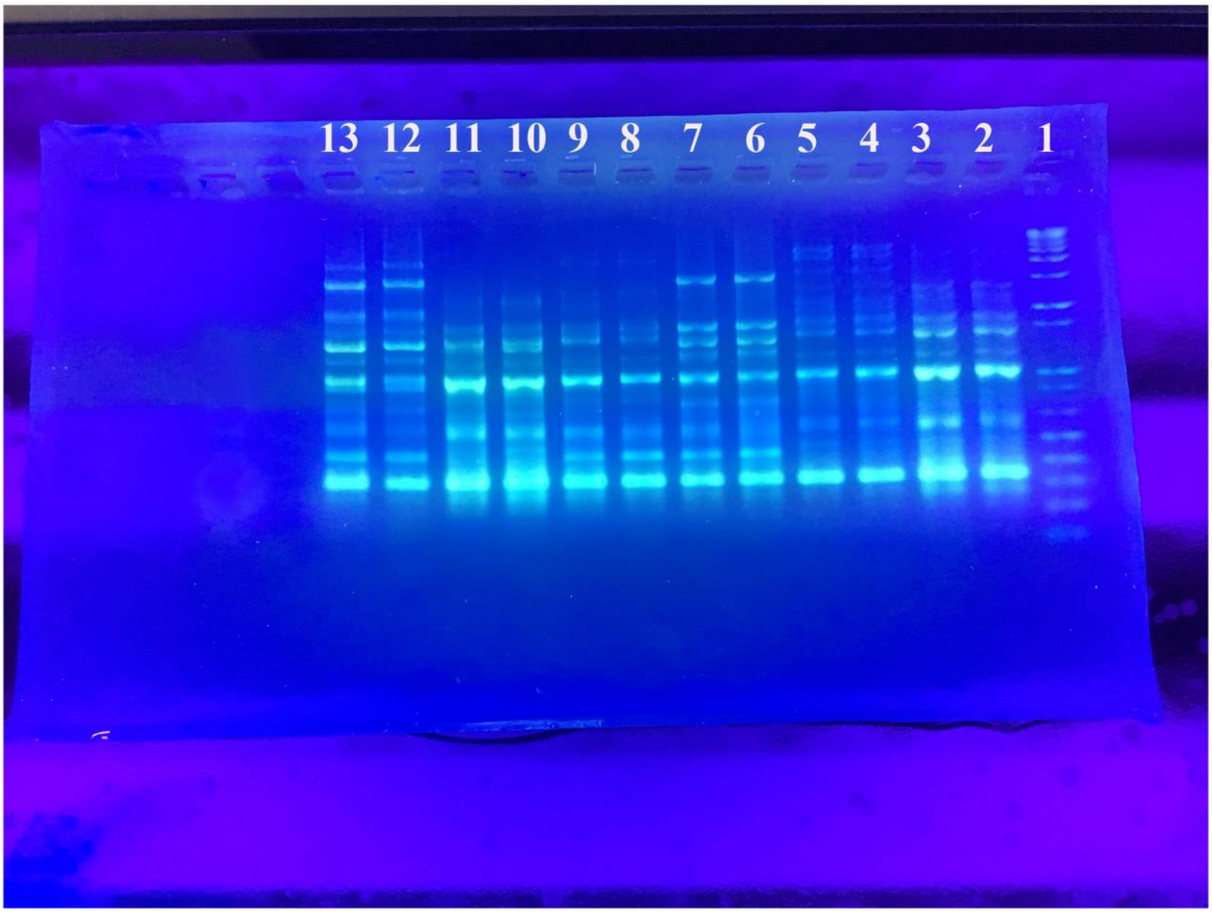
13 12 11 10 9 8 7 6 5 4 3 2 1

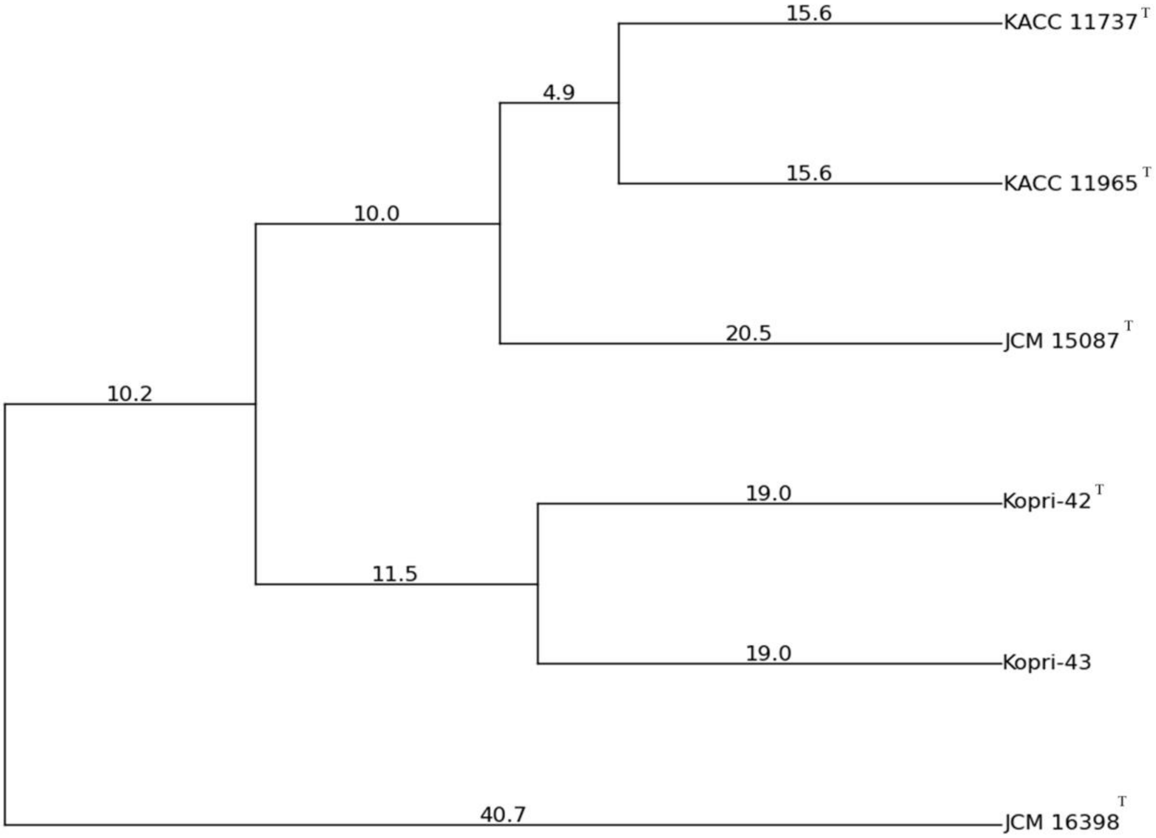
15.6
KACC 11737 T
4.9
15.6
KACC 11965 T
10.0
20.5
JCM 15087 T
10.2
19.0
Kopri-42 T
11.5
19.0
Kopri-43
40.7
JCM 16398 T

Fig. S1. REP-PCR band and dendogram detected after performing PCR amplification and gel-electrophoresis.

The gel-electrophoresis image was analyzed using PyElph 1.4 software. The dendogram illustrated below was generated using UPGMA method, with a bootstrap of 100. The similarity matrix was computed using Dice coefficient and the distance matrix have been displayed on the branches of the dendogram. Lanes: 1= 1 kb ladder; 2, 3: Kopri-42^{T}; 4, 5: Kopri-43; 6, 7: *F. psychrolimnae* KACC 11737^{T}; 8, 9: *F. limicola* KACC 11965^{T}; 10, 11: *F. sinopsychrotolerans* JCM 16398^{T}; 12, 13, *F. tiangeerense* JCM 15087^{T}.

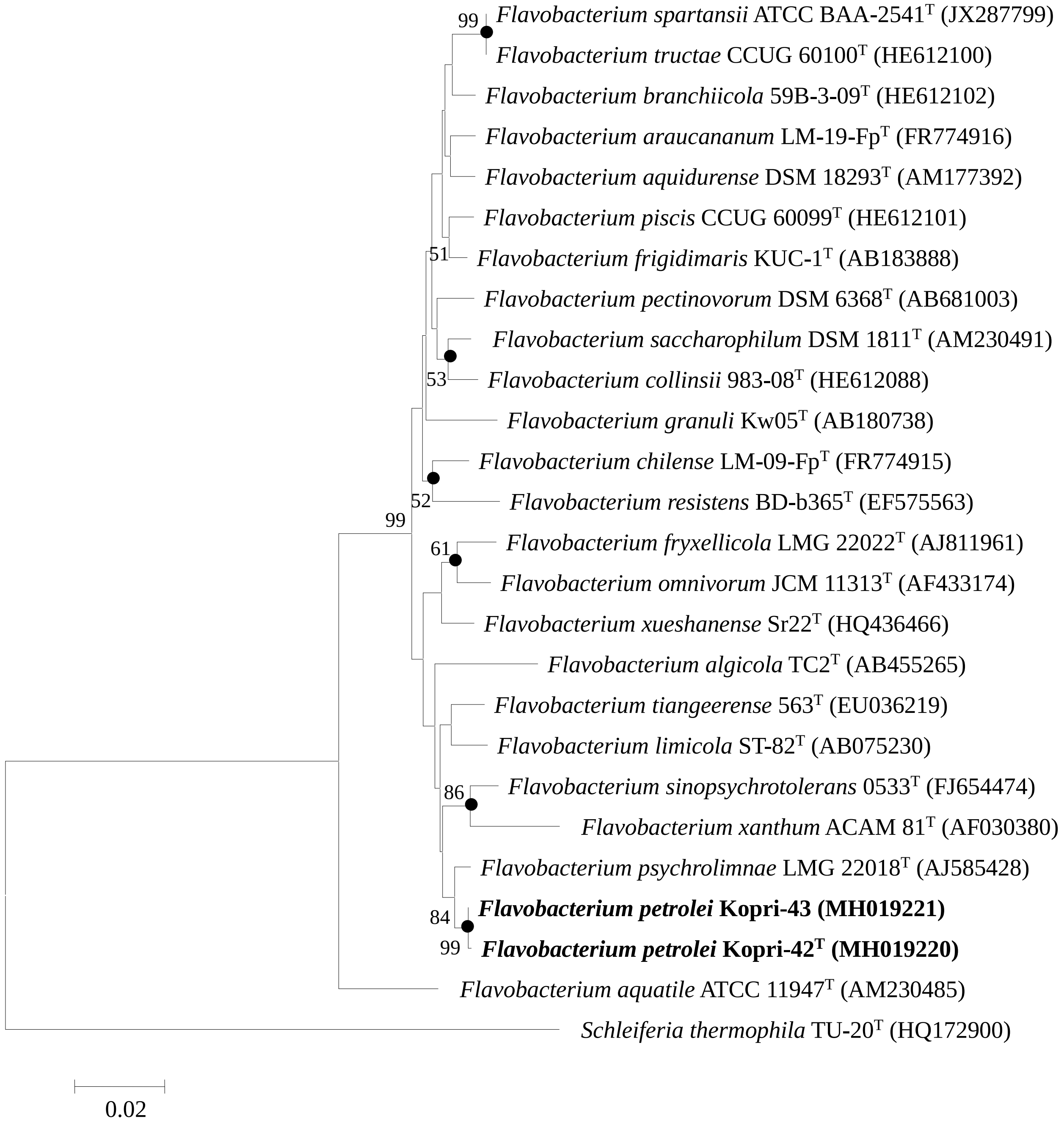

Fig. S2. Phylogenetic tree inferred with neighbor-joining algorithm based on 16S rRNA gene sequences showing the relationship between strains Kopri-42^{T}, Kopri-43, and closely related members of the genus *Flavobacterium*. Filled circles indicate branch-nodes recovered by neighbor-joining, maximum-likelihood, and maximum-parsimony phylogenetic trees. The numbers at the branch-nodes represent the percentage of 1,000 bootstrap replicates; only values >50% are depicted in the tree. GenBank accession numbers for 16S rRNA gene sequences are presented in parentheses. *Schleiferia thermophila* TU-20^{T} was included as an out-group. The scale bar represents 0.02 substitutions per nucleotide position.

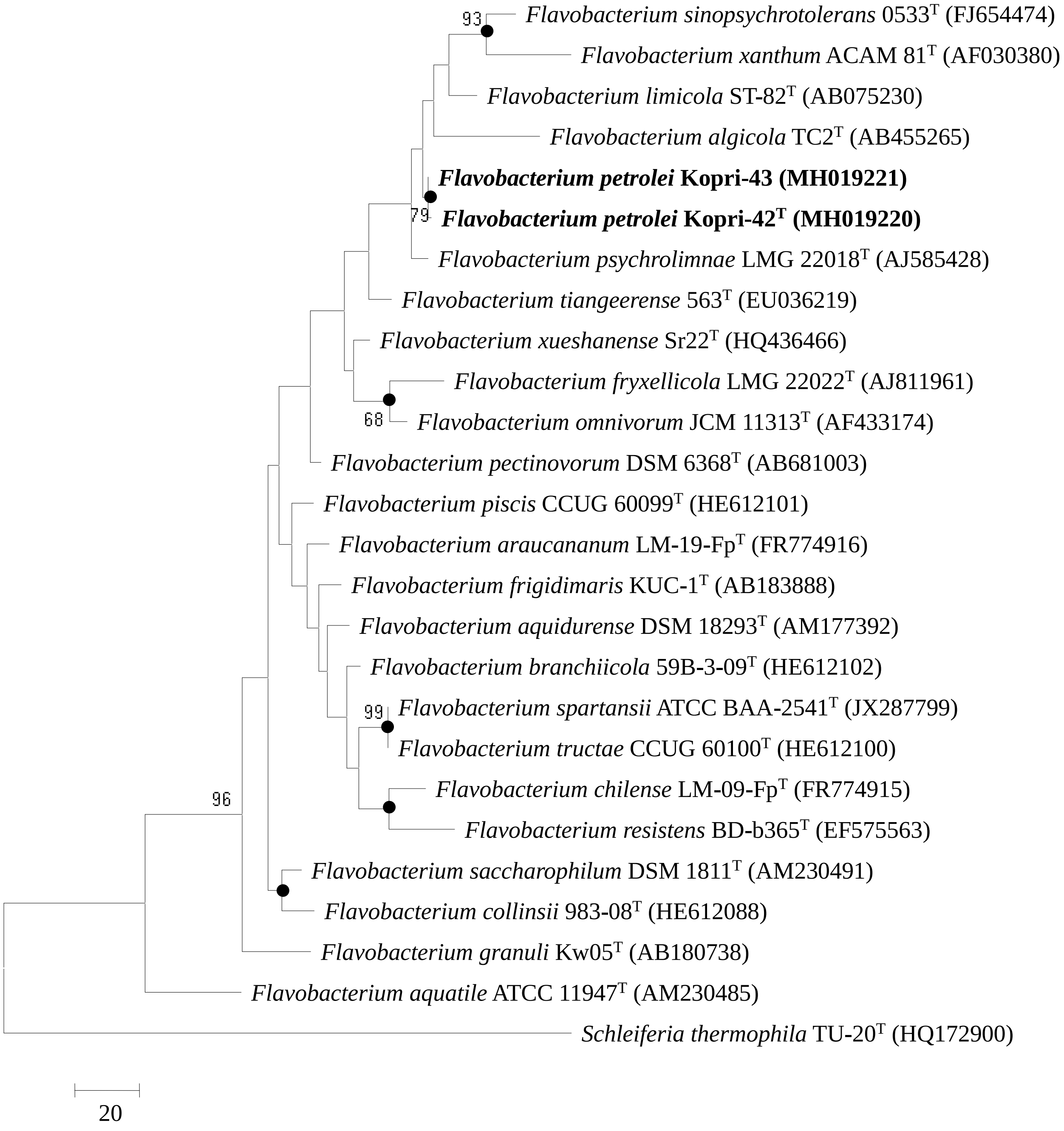

Fig. S3. Phylogenetic tree inferred with maximum-parsimony algorithm based on 16S rRNA gene sequences showing the relationship between strains Kopri-42^{T}, Kopri-43, and closely related members of the genus *Flavobacterium*. Filled circles indicate branch-nodes recovered by maximum-parsimony, maximum-likelihood, and neighbor-joining phylogenetic trees. The numbers at the branch-nodes represent the percentage of 1,000 bootstrap replicates; only values >50% are depicted in the tree. GenBank accession numbers for 16S rRNA gene sequences are presented in parentheses. *Schleiferia thermophila* TU-20^{T} was included as an out-group. The scale bar represents 20.0 substitutions per nucleotide position.

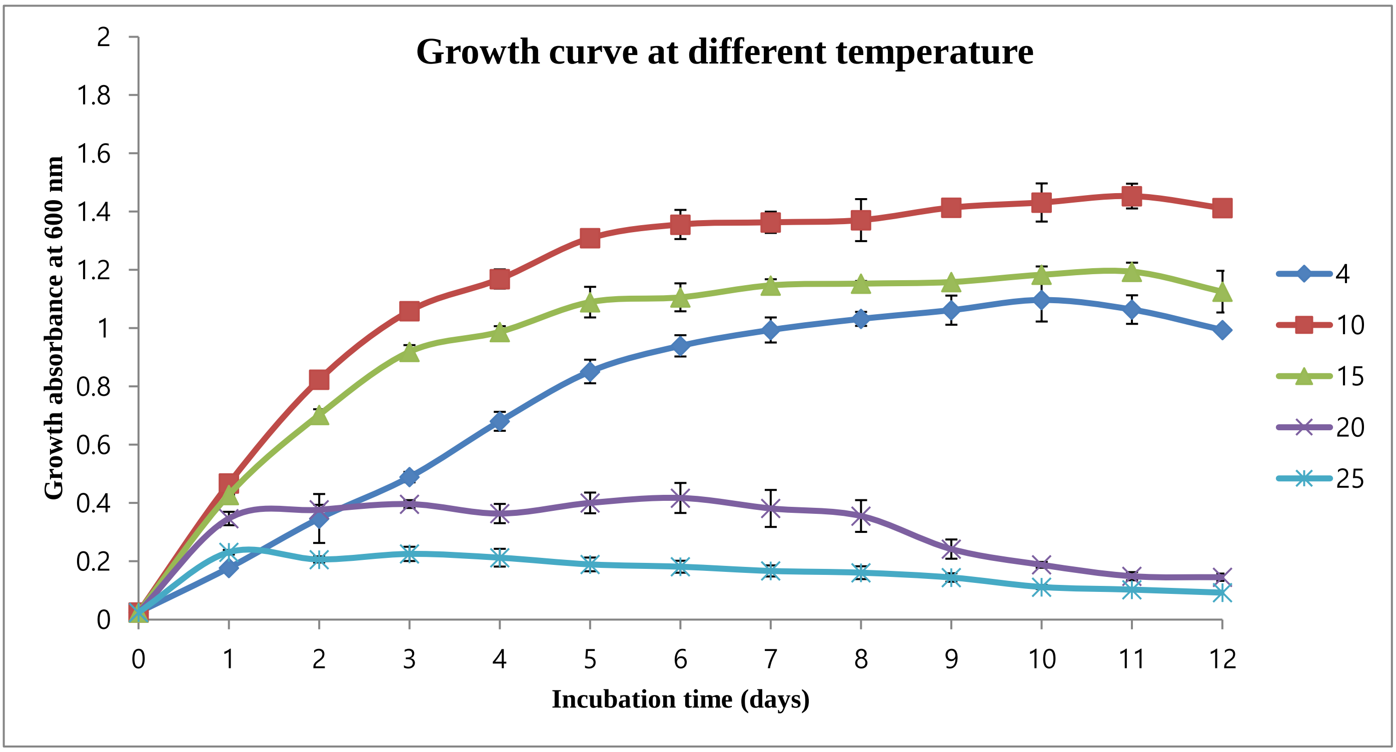

Fig. S4. Growth curve of strain Kopri-42^{T} constructed after measuring growth absorbance at 600 nm. The optimum temperature was determined by cultivating bacteria at temperatures 4, 10, 15, 20, and 25 °C.

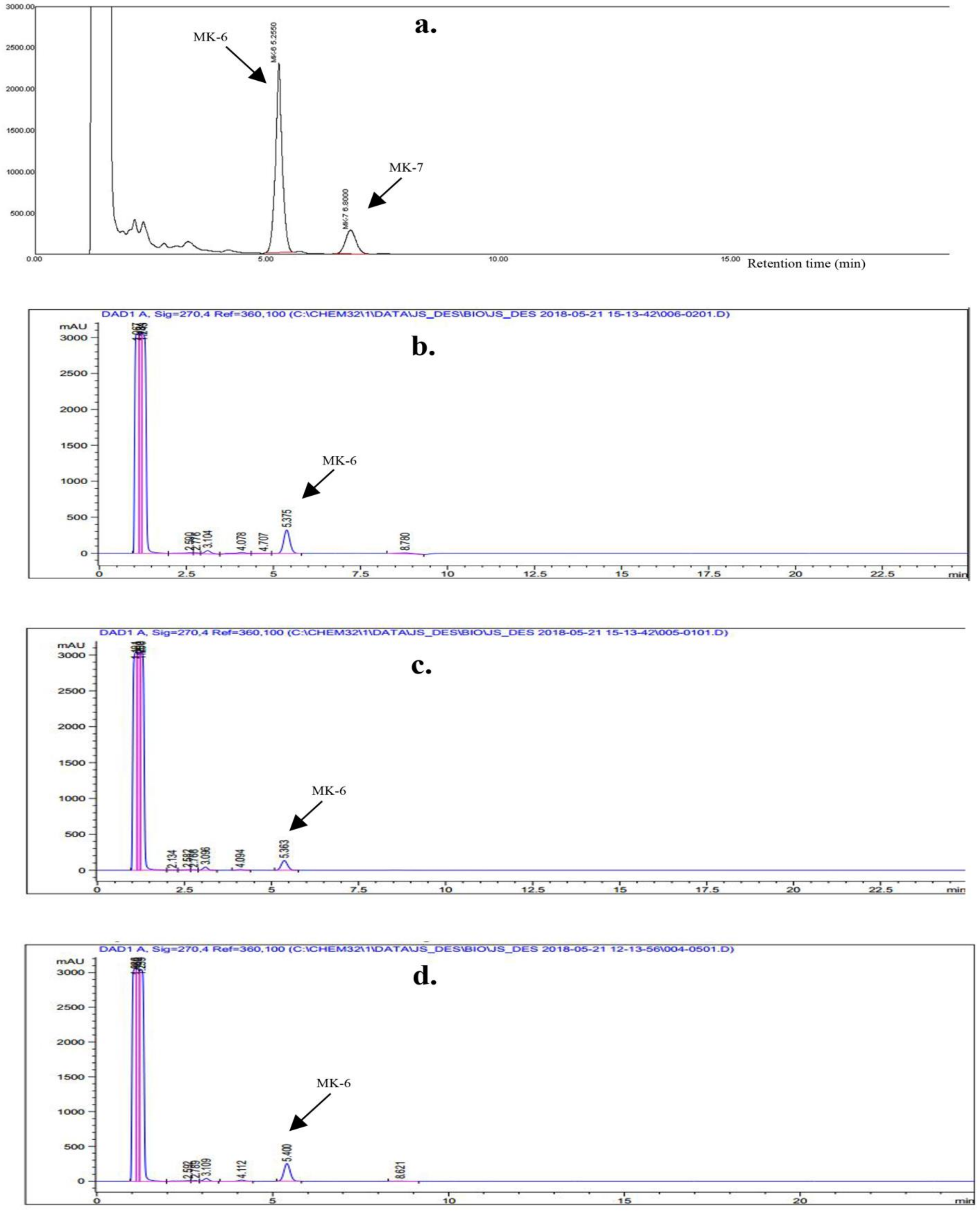

Fig. S5. Chromatograms of respiratory quinone detected by HPLC analysis after extracting from freeze-dry cells. a, standards; b, KACC 11737[T]; c, Kopri-43; and d, Kopri-42[T].

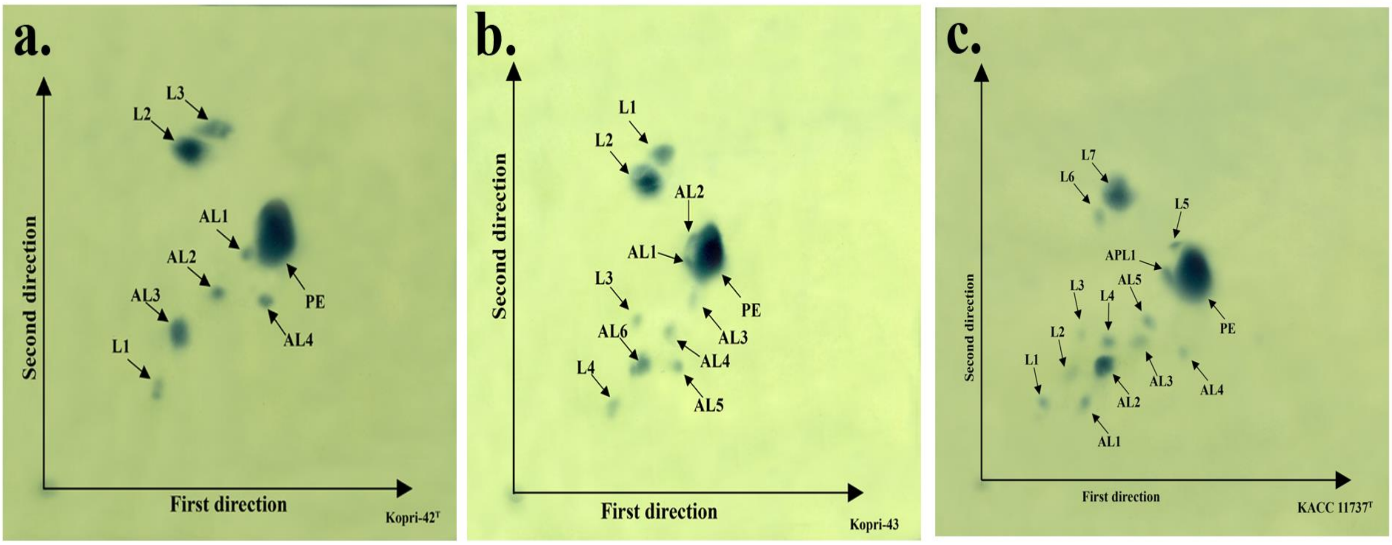

Fig. S6. Polar lipids profiles depicted from two-dimensional thin-layer chromatograms of strains: a) Kopri-42^{T}, b) Kopri-43, and c) KACC 11737^{T} detected with 5% w/v ethanolic molybdophosphoric acid reagent. Abbreviations: PE, phosphatidylethanolamine; AL1-AL6, unidentified aminolipids; APL1, unidentified aminophospholipid; L1–L7, unidentified polar lipids.

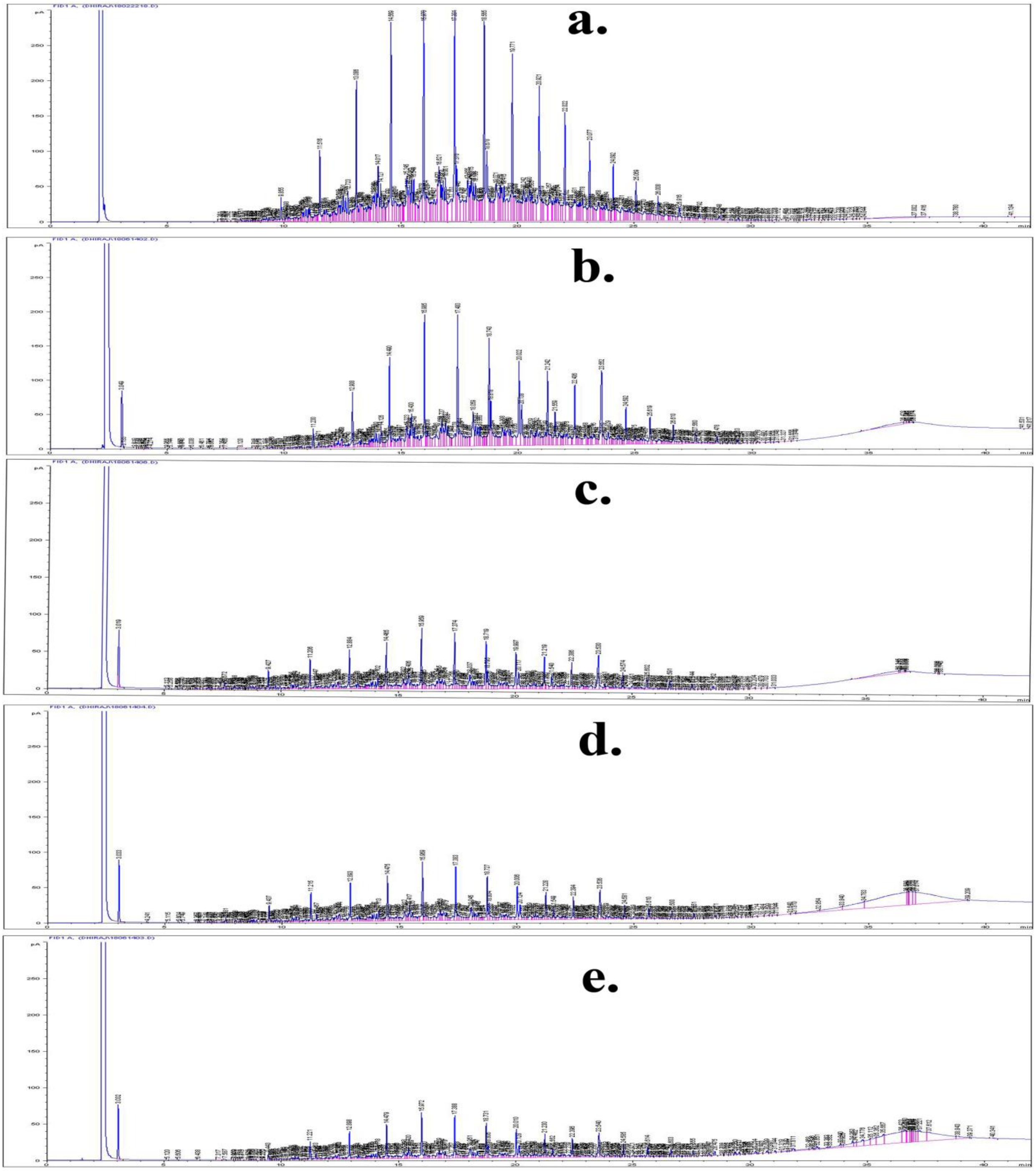

Fig. S7. GC-FID Chromatograms of residual diesel oil in different treatment conditions analyzed after 30-day experiment. a, control; b. Kopri-42; c, Kopri-42 + nutrients; d, Kopri-42 + biosurfactants; and e, Kopri-42 + nutrients + biosurfactants.
